# The construction of the evaluation system of nurses' post‐training and the application of the system in 25 grade‐A general hospitals in China

**DOI:** 10.1002/nop2.651

**Published:** 2020-10-14

**Authors:** Linlin Jiao, Yuanda Sui, Guihua Yang, Pulin Wang, Qiaofeng Li, Junhong Chen, Lili Liu, Chunling Yang

**Affiliations:** ^1^ Key Laboratory of Clinical Nursing Nursing Department Liaocheng People's Hospital Liaocheng China; ^2^ Department of Critical Care Medicine Liaocheng People's Hospital Liaocheng China; ^3^ Nursing Department Liaocheng People's Hospital Liaocheng China; ^4^ Booking Information Center Liaocheng People's Hospital Liaocheng China; ^5^ Breast Center Liaocheng People's Hospital Liaocheng China; ^6^ Internal Medicine‐Cardiovascular Department Liaocheng People's Hospital Liaocheng China

**Keywords:** evaluation research training, nurses, nursing

## Abstract

**Aims:**

To make the evaluation more scientific, structured and systematic, this study aims to develop an evaluation index system for nurses training and to explore clinical effect of system.

**Design:**

The evaluation index system of nurses’ post‐training was constructed using the Delphi method.

**Methods:**

Introducing the system, we used the pre‐work training of new nurses as an example for discussing the specific implementation scheme of the system. Twenty‐five tertiary and first‐class general hospitals in 14 provinces and municipalities were evaluated on the spot, and the application effect of the system was evaluated comprehensively.

**Results:**

The index system consisted of three first‐grade indexes, seven second‐grade indexes and 23 third‐grade indexes. There were three levels in teaching and training ability, and the distance had statistical significance.

## 1 INTRODUCTION

Nurses account for approximately 0.005% of the total population in most countries of the world, while they count for only 0.001% in China. The shortage of nurses has become a common problem faced by hospitals all over China, especially large third‐class general hospitals. For this reason, hospitals, clinics, community service stations and other major hospitals have vigorously introduced nursing talents to make up for the shortage of nurses. According to the National Nursing Development Plan of 2016, the number of Registered Nurses in China will reach 4.45 million by 2020 and the comprehensive quality and professional and technical ability of nurses will directly affect the quality of clinical nursing and patient safety. With the transformation of the medical model and the continuous advancement of the medical and health system reforms, the people pose higher requirements for the quality and ability of nurses. Nurse post‐training is an important way to maintain and expand nurses’ abilities and to improve the professional quality of nurses. It provides nursing personnel a guarantee for maintaining and improving people's health (Lewinson et al., 2015; Okoli et al., 2015; Stirling, 2015). The question of how to train nurses effectively has become an urgent one among nursing managers in hospitals. Although nurses' on‐the‐job training is widely carried out in China – often with a great deal of money and energy invested – there is no evidence to support whether this training can bring enough benefits to patients, nurses and hospitals. Training evaluation is of great significance for ensuring training effectiveness, measuring training performance and achieving training objectives (Janssens & Van Amerlsvoort, 2008; Mehralizadeh et al., 2007). Nemec (2018) pointed out that most health institutions do not evaluate training at all or that they limit their evaluation to simple satisfaction indicators in the United States. South Africa and other countries use only a self‐report feedback scale and 360 degree to measure the training evaluation work (Mash et al., 2018). Scholars have pointed out that understanding the cost and benefit of training is very important to ensure the monetary value of hospital capital input; the economic evaluation of training is very limited and it is necessary to expand and standardize the process and to select an appropriate economic evaluation model to capture the cost and benefit of training to achieve comparability of training effectiveness in different environments (Banke‐Thomas et al., 2017). However, the training evaluation of nurses in China also faces complex challenges. Without a thorough understanding of the essence of the training system cycle, the whole process of training and the results of training at all levels have not been fully excavated in time and space. The nursing training system is inefficient. There is a lack of evaluation of training, a lagging behind of evaluation results, there are obstacles encountered in training migration, a lack of strategic orientation in evaluation activities, a single evaluation method and a lack of long‐term follow‐up evaluation mechanism, etc. Therefore, it is particularly urgent that we do a good job in evaluating the effect of nurses' on‐the‐job training. The purpose of this study is to construct a scientific and reasonable evaluation index system of nurse post‐training and to explore the clinical effect of the system, so as to provide a theoretical basis for enriching the system of training and improving the evaluation of hospital nurse post‐training.

## 2 METHODS

### 2.1 The construction process of the evaluation index system

#### 2.1.1 The establishment of a research group

A research group was established in November 2015. The research group consisted of eight members, including two nursing education management experts, four clinical managers and two nursing master's degree students. Among them, the nursing education management experts were responsible for supervising and inspecting the research process and providing manpower and material support; the nursing postgraduates were responsible for the whole research design, data processing, statistical analysis and carrying out the clinical practice of the index system; the clinical managers were responsible for forming semi‐structured interview outlines, formulating interviewees, forming expert inquiry papers, selecting experts and completing three rounds of inquiry workers.

#### 2.1.2 The preliminary selection of evaluation indicators

According to the requirements of the State Council of the People's Republic of China's National Plan for Medical and Health Services System (2015–2020) (No. 14 issued by the State Office [2015]), the Opinions of the State Council of the Central Committee of the CPC on Deepening the Reform of Medical and Health System, the Outline of the "Healthy China 2030" Plan and the "Thirteenth Five‐Year Plan for Health and Health" (No. 77 issued by the State Council), the evaluation of nurses' post‐training is a significant issue. Emphasis should be put on strengthening the construction of nursing professionals, improving the talent evaluation mechanism, improving the overall service ability and level of clinical specialty and promoting the professional development and workability of nurses, etc. Meanwhile, reference should be made to relevant domestic and international literature to determine the evaluation subject category of nurses' training effect. The present study chooses seven secondary indicators including organizational management, training programme, training environment, training teaching, training organization, training content mastery and radiation effect on the organization.

#### 2.1.3 The establishment of the evaluation model

Some scholars have exploratorily introduced evaluation models to guide the evaluation (Moore et al., 2009; Smidt et al., 2009). The classical training evaluation models belong to the Kirkpatrick model, Kaufman five‐level model, Philips five‐level ROI framework model, CIRO model and CIPP model, etc. (Kaufman & Kelley, 1994; Kirkpatrick, 1996, 2009; Phillips, 1995). CIPP and the Kirkpatrick model were the most widely used training theoretical models. CIPP includes four parts: context, input, process and product. Not only does it analyse the necessity and feasibility of training, but it also pays attention to the monitoring of the whole training process. The Kirkpatrick model includes four progressive levels: the reflection level, the learning level, the behaviour level and the result level. The Kirkpatrick model focuses primarily on the evaluation of training results and secondarily on the concretization of CIPP model results. From December 2015–January 2016, CIPP and the Kirkpatrick model were combined organically from the perspective of training management. Considering the training's lag, ambiguity and poor segmentation characteristics and the matching of the hospital performance management background, a set of education and training evaluation models suitable for the development of China's national conditions was constructed. The evaluation model can be roughly divided into three levels: pre‐training evaluation, that is background and input level of CIPP model; the training process evaluation, that is the process level of the CIPP model; and post‐training evaluation, that is the four levels of Coriolis model (concretization of the CIPP model output evaluation).

#### 2.1.4 The formulation of expert inquiry papers

1. Searching the literature: From December 2015–March 2016, literature retrieval was conducted using the China National Knowledge Infrastructure, the Wanfang Database, PubMed and Web of Science, etc. by searching for key words such as “training,” “index system” and “evaluation” to collect all the evaluation indexes of nurse training.
2. Expert interviews: In April 2016, 19 nurses, training managers and nursing educators were selected as interviewees to partake in semi‐structured interviews. Before the interview, team members were trained in a unified face‐to‐face manner, clarifying the purpose of the interview, the topic of the interview, the inclusion criteria of the interviewees, the time of the interview, the matters needing attention in the process of the interview implementation, etc. These took the form of one‐to‐one, face‐to‐face interviews, each lasting approximately 30 min. The main themes were "Do you think it is necessary to evaluate the effect of training after it has taken place?" and "What suggestions do you have for setting up the evaluation index of nurses' on‐the‐job training?" Experts revised and perfected the index categories, names, definitions, connotations and calculation methods one by one and initially formulated the framework of evaluation index system for nurses' job training.
3. Pre‐Survey: Based on semi‐structured interviews and a literature review, a small‐scale pre‐survey was carried out according to the framework of the evaluation index system for nurse post‐training. Six experts of nursing management, nursing education, medical education and clinical nursing were invited to discuss and revise the structure level, classification and expression of indicators in the framework.
4. According to the preliminary investigation, an expert inquiry questionnaire was finally formed, which consisted of three parts: 1) completing the form; 2) including index name, index connotation, calculation formula, Likert grade 5 score and revision opinion column; and 3) Basic situation of experts: general information such as age, length of service, professional title, educational background and expert's familiarity with research issues and judgement basis.

#### 2.1.5 Expert selection and inquiry

**Figure 1:**
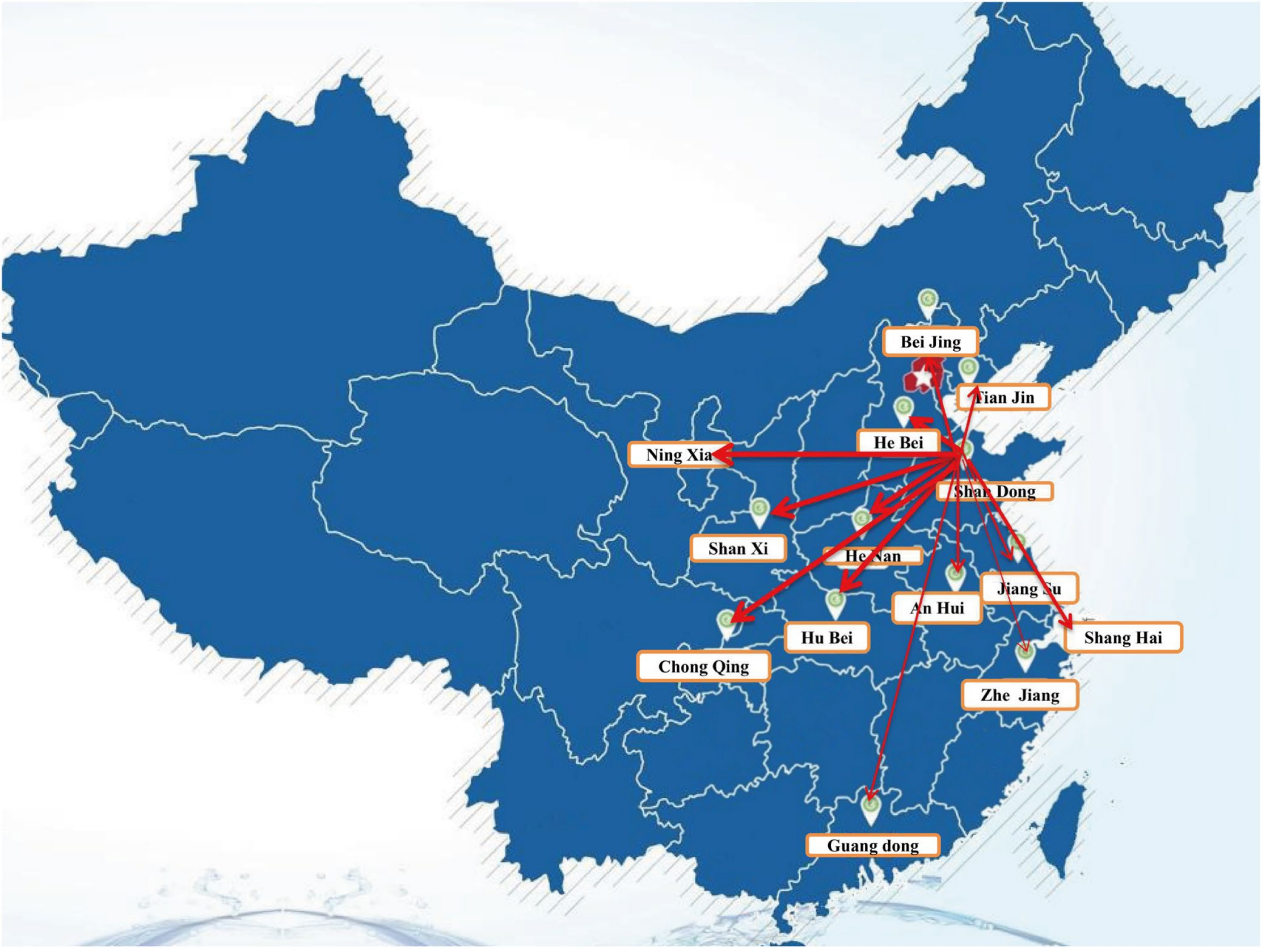
The Distribution of 32 experts across the 14 provinces and municipalities screened

Expert filtrating: In June 2016, 32 specialists were selected from Shandong, Beijing, Tianjin, Henan, Hebei, Shanghai, Guangdong, Zhejiang, Anhui, Hubei, Chongqing, Shaanxi, Ningxia and Jiangsu provinces or cities from 17 tertiary‐A hospitals or nursing colleges in key universities in China. (Figure 1) Participants were selected if they had a bachelor's degree or above, held a post of associate senior or associate professor or above, were responsible for nursing post‐training management or nursing teaching for more than 15 years, were highly motivated and were willing to cooperate in the completion of multiple rounds of expert consultation. The first two rounds of questionnaires were distributed to 32 consultants, while the third round consisted of 31 consultants, as one withdrew due to illness. There were nine senior and 23 vice senior titles, 11 master's degrees and 21 undergraduate students, aged 38–57 (46.17 *SD* 2.12) with working years from 15–37 (19.28 *SD* 3.23).

#### 2.1.6 Constructing the index system

##### The establishment of the evaluation index using the Delphi method

From July–October 2016, the inquiry volume was sent to experts by mail and the inquiry explanation and confirmation were provided via telephone with experts. In the first two rounds of expert consultation, 32 questionnaires were sent out and 32 questionnaires were recovered. The initial response rate of expert questionnaires was 100% and that of the third round was 96.88%. According to the results of three rounds of expert inquiries, the second and third evaluation indicators were determined.

##### Determining the weight of indicators using the analytic hierarchy process

In the study, the evaluation index system was composed of the first‐, second‐ and third‐level indicators, of which there were seven secondary indicators and there were several three‐level indicators under each secondary indicator. First, the seven secondary indicators were compared and a grade was assessed according to their importance, using the pair comparison method and a 1–9 comparative scale to create a comparison matrix; the same method was then used to construct a comparison matrix for the three‐level indicator under each secondary indicator.

For the second‐ and third‐level indicators, the maximum eigenvalues and corresponding eigenvectors were calculated, respectively. At the same time, a consistency test was carried out. When the random consistency ratio is less than 0.1, it can be concluded that the weight obtained by the judgement matrix meets the consistency requirement (Ishizaka & Labib, 2004; Satty, 1980, 2006; Taleai & Manaourian, 2008).

### 2.2 Implementing the process of evaluation index system

To further validate the feasibility of the evaluation index system, the research group conducted in‐depth analysis and discussion of the previous research results and formulated the evaluation criteria and rules of the system combined with the established evaluation index system of nurses' post‐training. A total of 169 new nurses from a tertiary‐A general hospital in August 2017 were selected as participants for a six‐month pre‐job training programme. The time of evaluation can be divided into three stages: pre‐training evaluation, training process evaluation and post‐training evaluation; this was to explore the specific implementation plan of the system. To enhance the standardization and effectiveness of the training and to avoid the influence of subjective factors, the whole evaluation was carried out by the members of this subject group. According to the evaluation criteria and methods of the system, the corresponding checklist or questionnaire or examination paper were designed and embedded in the Nursing Information System platform or 512 examination education network. The whole evaluation process was carried out in the information system, including data entry, extraction, statistical analysis and other functions.

#### 2.2.1 Pre‐training assessment

Before the training of new nurses, first, we held a symposium for nursing managers to clarify whether each department had formulated training programmes and plans for new nurses, whether the title, educational background, teaching age and professional level of the teaching staff were reasonable, the division of training for the instructors and the training methods used (combination of scenario simulation and classroom teaching, PBL, group demonstration, discussion, network learning), etc. Second, the theoretical knowledge and relevant operational skills of new nurses were investigated using self‐designed test papers and the training goal was established by investigating the training needs of new nurses with the questionnaire of pre–post training needs.

#### 2.2.2 The training process assessment

New nurses were trained for 3 months. The three‐level index C10‐C13 was used as the evaluation index of training process. The evaluation tools were as follows: (1) a self‐designed pre‐job training feedback questionnaire for new nurses was designed to understand the subjective feelings and evaluation of the whole training project of new nurses; and (2) a self‐designed Clinical Nursing Teaching Training Plan Implementation Evaluation Table to extract the implementation rate, ensuring a timely understanding of the progress of training, so as to amend and improve the follow‐up training projects.

#### 2.2.3 Post‐training assessment

Post‐training assessment is divided into immediate evaluation, short‐term evaluation and long‐term evaluation:

1. Immediate evaluation: At the end of pre‐job training for new nurses, it can be used to judge the degree of the realization of the training objectives, the satisfaction of the nurses and the improvement of nurses' knowledge and skills. The three‐level indicator C14‐C17 is the immediate evaluation index, and the evaluation tools were as follows: the feedback questionnaire of pre‐job training for new nurses (self‐designed); the theory and operation examination; and the comprehensive ability assessment form at bedside (self‐designed).
2. Short‐term effect assessment was conducted 3–6 months after the completion of pre‐job training for new nurses. It was used to assess whether the knowledge and skills learned by nurses in training had been applied in the workplace and the degree of improvement of their work. The three‐level indicator C18‐C21 is the short‐term evaluation index. The evaluation tools were all self‐designed evaluation tables: the Nurse Humanistic Care Implementation Evaluation Form; the Nurse Behavior Standard Implementation Evaluation Form; the Nurse Work Attitude Evaluation Form; the Nurse Clinical Nursing Ability Evaluation Form; the Nurses' clinical decision‐making and emergency response ability evaluation form; self‐designed nurse–patient Communication Evaluation Form; self‐designed Nursing Adverse Event Management Standard implementation checklist; checklist for goods and equipment management standard; checklist for Management Code of first‐aid drugs, articles and instruments; checklist of Drug Administration regulations; checklist of nursing document management norms; Implementation checklist for prevention of catheter‐related bloodstream infection; Standard practice for thrombosis preventive care; checklist for standardized implementation of preventive measures for ventilator‐associated pneumonia Implementation; checklist of nursing measures for tracheal intubation; checklist for nursing care of patients at high risk of falls; checklist for nursing care of high‐risk patients with stress injury, etc.
3. The long‐term effect evaluation is conducted more than half a year after the completion of pre‐job training for new nurses. It evaluates the long‐term to of training programmes on nurses and organizations. Three‐level indicators C22 and C23 are long‐term evaluation indicators. The evaluation tools were a 360‐degree evaluation (self‐designed), patient complaint rate (number of complaints/number of hospitalized patients *100%), nurse–patient dispute rate (number of nurse–patient disputes/number of hospitalized patients *100%), adverse events and patient satisfaction questionnaire (Labarere et al., 2001).

### 2.3 The application effect of the evaluation index system

**Figure 2:**
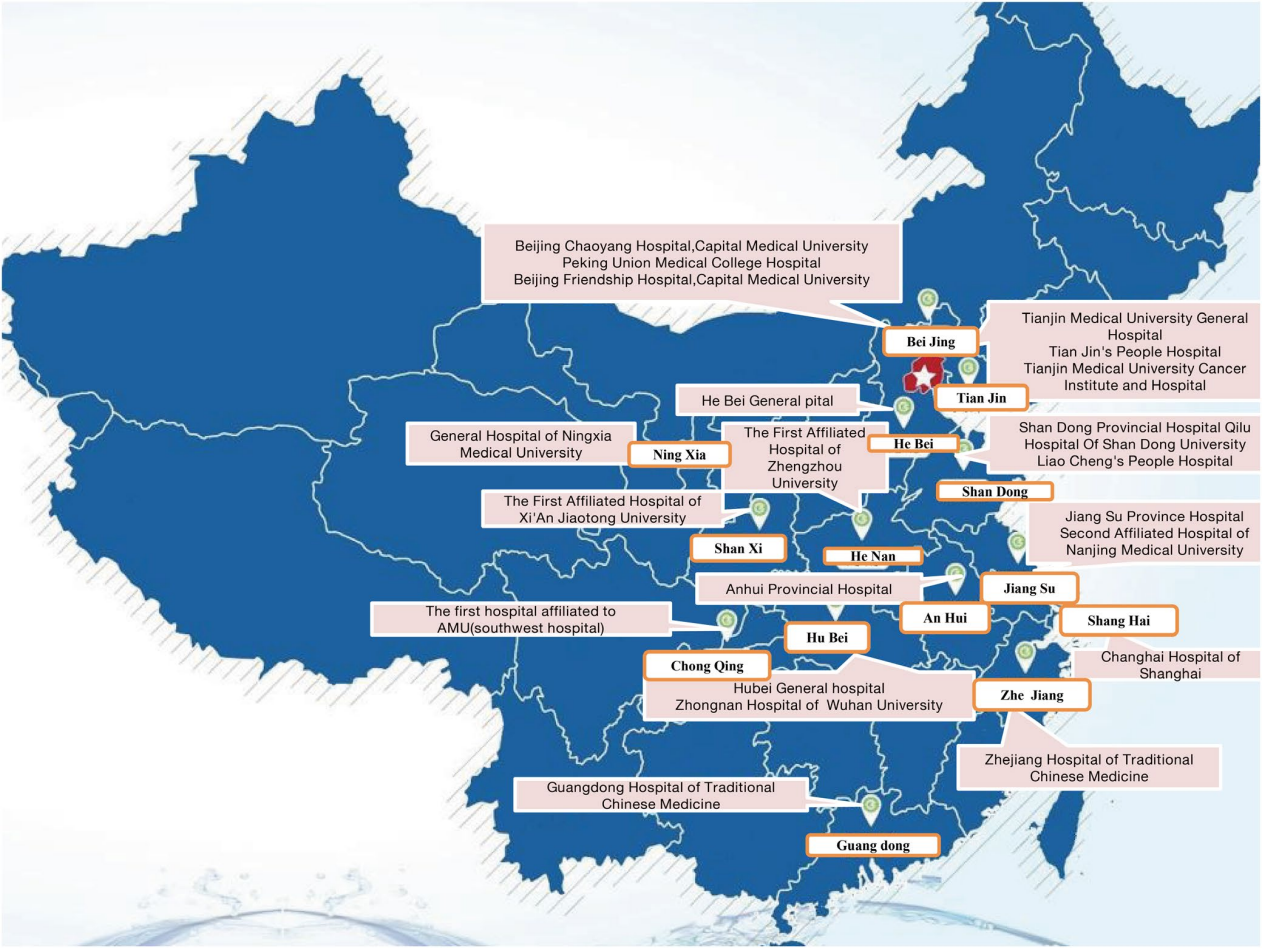
The Distribution of the 25 tertiary and first‐class general hospitals across 14 provinces and municipalities

To verify the application effect of the evaluation index system, from November 2016–September 2018, our team selected 25 general hospitals in 14 provinces and municipalities in China for evaluation, which were represented by the letters A~Y ( Figure 2). We strictly abided by the procedures and conditions required by our methodologies and controlled the admission criteria of hospitals as follows: all hospitals were grade three and grade‐A general hospitals; we obtained the approval of the hospital ethics committee; we excluded hospitals that refused to participate in research; we selected hospitals where experts participated in three rounds of expert inquiries; and we distributed our study as far as possible across hospitals of all provinces and cities throughout the country.

Before conducting an on‐site evaluation of 25 hospitals, hospital experts who were familiar with nurses' job training were selected. The members of the task group contacted them by telephone to explain the purpose, content and method of the investigation, to obtain their consent and to collect the basic information of investigators (name, phone number, gender, title, etc.). The basic information of the hospital is shown in Table 1. The investigators of the 25 hospitals were trained online by specialists. They were required to master the connotations of the evaluation index system, the evaluation methods and the application of evaluation tools. If they had questions, they were required answer questions online in time, emphasizing anonymity and confidentiality of information. The investigators organized and carried out the investigation according to the unified procedure established during the training and completed the investigation within six months. Members of the research group supervised the investigation of the hospital at all times via telephone, WeChat®, etc. After the survey, the investigators sent the original data back to the researchers. After receiving the questionnaire, the researchers checked the completeness of the answers one by one and numbered them.

**Table 1:** The basic situation of 25 third‐grade class A general hospitals investigated

Hospital Name	Hospital Level	Survey Method	Number of Surveyors	Subjects of investigation
A hospital	A class tertiary hospital	Nursing Information System	59	Intensive care talent pool
B hospital	A class tertiary hospital	Nursing Information System	41	Mobile Nurse Warehouse
C hospital	A class tertiary hospital	Nursing Information System	49	Training of newcomer nurses
D hospital	A class tertiary hospital	Nursing Information System	74	Emergency unit
E hospital	A class tertiary hospital	Printed paper	66	Specialized nurses
F hospital	A class tertiary hospital	Nursing Information System	37	Nursing managers
G hospital	A class tertiary hospital	Nursing Information System	38	Nursing Instructor
H hospital	A class tertiary hospital	Nursing Information System	58	Intensive care talent pool
I hospital	A class tertiary hospital	Nursing Information System	55	Mobile Nurse Warehouse
J hospital	A class tertiary hospital	Printed paper	29	training of newcomer nurses
K hospital	A class tertiary hospital	Printed paper	33	training of newcomer nurses
L hospital	A class tertiary hospital	Printed paper	56	training of newcomer nurses
M hospital	A class tertiary hospital	Printed paper	47	Specialized nurses
N hospital	A class tertiary hospital	Printed paper	37	Intensive care talent pool
O hospital	A class tertiary hospital	Printed paper	62	Mobile Nurse Warehouse
P hospital	A class tertiary hospital	Nursing Information System	38	Emergency unit
Q hospital	A class tertiary hospital	Nursing Information System	49	Emergency unit
R hospital	A class tertiary hospital	Nursing Information System	34	Nursing managers
S hospital	A class tertiary hospital	Nursing Information System	41	Nursing Instructor
T hospital	A class tertiary hospital	Nursing Information System	14	Nurse for further study
U hospital	A class tertiary hospital	Nursing Information System	29	Nurse for further study
V hospital	A class tertiary hospital	Printed paper	9	Nurse for further study
W hospital	A class tertiary hospital	Printed paper	59	Specialized nurses
X hospital	A class tertiary hospital	Printed paper	48	Internship Nursing students
Y hospital	A class tertiary hospital	Printed paper	31	Internship Nursing students

### 2.4 Data collection and statistical analysis

SPSS19.0 was used to calculate experts' enthusiasm, authority and coordination coefficients, to test the reliability of experts and AHP9.0 statistical software was used to calculate the weight of indicators. Metrological data were expressed by mean and standard deviation, and counting data were expressed in examples and percentages. SPSS19.0 was used for cluster analysis and variance analysis of the comprehensive score of hospital training level; *p < *.05 was considered statistically significant.

## 3 RESULTS

### 3.1 Result of expert inquiry

#### 3.1.1 The enthusiasm of experts

The enthusiasm of experts was expressed by the response rate of the questionnaire. In the first round of expert inquiry, 32 questionnaires were sent out, 32 valid questionnaires were recovered, and the recovery rate was 100%. In the second round, 32 valid questionnaires were sent out and the recovery rate was 100%. In the third round, 32 valid questionnaires were sent out and 31 valid questionnaires were recovered, with an effective recovery rate of 96.88%.

#### 3.1.2 The authority of experts

The degree of authority of experts is the arithmetic average of the sum of the judgement basis and familiarity of the indicators in the expert self‐evaluation table, which was expressed by Cr. In the study, the Cr of consultation experts was 0.82.

#### 3.1.3 Degree of coordination of expert opinions

The coordination degree of expert opinions was generally expressed by a coordination coefficient. The coordination degree of the first, second and third round of consultation expert opinions was 0.889, 0.847 and 0.869, respectively. Chi‐square test showed that there was a statistical significance (*p* < .01).

#### 3.1.4 Revision, weight and consistency test of evaluation indicators at all levels

After expert inquiry and statistical analysis of the data, the experts agreed on the second‐level indicators without any modification; in the third‐level indicators, 3 items were merged, 7 items were modified, and 13 items were deleted. Taking the secondary index as an example, the judgement matrix was constructed and the weights of each index were normalized (Table 2). The consistency test of the judgement matrix is carried out (Table 3) and Cr < 0.1. It could be concluded that the weights obtained by the judgement matrix meet the consistency requirements.

**Table 2:** The Judgement Matrix and Weight of Level II Indicators

Index	Organizational Management	Training Programmes	Training Environment	Training Teaching	Training Organization	Mastery of Training Contents	Radiation effects on tissues	weight
Organizational Management	1	6/7	2/7	3/13	3/13	7/35	7/35	0.36
Training Programmes	7/6	1	3/9	8/32	8/32	3/13	3/13	0.335
Training Environment	7/35	7/35	1	5/15	5/15	8/32	8/32	0.115
Training Teaching	3/13	3/13	3/13	1	3/11	5/15	5/15	0.098
Training Organization	8/32	8/32	8/32	11/3	1	3/11	3/11	0.048
Mastery of Training Contents	5/15	5/15	5/15	5/2	2/7	1	29/8	0.349
Radiation effects on tissues	3/11	3/11	3/11	3/8	3/9	3/9	1	0.059

**Table 3:** A consistency test of the judgement matrix

Matrix name	Matrix order	λmax	Ci值	Cr值
A‐B	5	5.194	0.0442	0.0478
B1‐C	7	8.154	0.0478	0.0514
B2‐C	8	7.139	0.0514	0.055
B3‐C	9	9.144	0.055	0.0586
B4‐C	6	6.558	0.0586	0.0622
B5‐C	5	3.972	0.0622	0.0658
B6‐C	4	1.386	0.0658	0.0124
B7‐C	3	4.953	0.0694	0.0438
B8‐C	2		0.073	

#### 3.1.5 Determining the evaluation index system

The evaluation index system was finally established, which consisted of three first‐level indicators, seven second‐level indicators and 23 third‐level indicators. At the same time, according to the experts' assignment of the importance of indicators at each level, the weight and coefficient of variation of indicators at each level were determined (Table 4).

**Table 4:** Weight, importance score and variation coefficient of nurse post‐training evaluation index system

Index	Weight (%)	Importance score（*x* ± *s*）	Coefficient of variation
A1 Pre‐training assessment	28.35	4.58 ± 0.54	0.11
B1 Organization and management	5.73	4.54 ± 0.55	0.13
A1‐B1‐C1 Policy and Planning	1.61	4.58 ± 0.62	0.14
A1‐B1‐C2 Teaching staff	4.12	4.71 ± 0.51	0.10
B2 Training plan	18.93	4.54 ± 0.57	0.12
A1‐B2‐C3 Training objectives	3.89	4.57 ± 0.61	0.15
A1‐B2‐C4 Training contents	4.92	4.52 ± 0.50	0.11
A1‐B2‐C5 Training plan	7.86	4.59 ± 0.37	0.11
A1‐B2‐C6 Outline and textbooks	2.26	4.44 ± 0.49	0.13
B3 Training environment	5.54	4.45 ± 0.63	0.13
A1‐B3‐C7 Training materials	4.20	4.49 ± 0.44	0.11
A1‐B3‐C8 Training ground	0.85	4.37 ± 0.46	0.10
A1‐B3‐C9 Training Instruments and Equipment	0.49	4.60 ± 0.57	0.13
A2 Training process assessment	18.67	4.43 ± 0.43	0.10
B4 Training teaching	18.68	4.18 ± 0.46	0.10
A2‐B4‐C10 Course Hours Arrangement	3.90	4.32 ± 0.53	0.13
A2‐B4‐C11 Training contents	7.84	4.46 ± 0.40	0.09
A2‐B4‐C12 Training teachers	3.29	4.27 ± 0.57	0.11
A2‐B4‐C13 Implementation of the plan	3.65	4.73 ± 0.70	0.15
A3 Post‐training assessment	52.98	4.79 ± 0.45	0.10
B5 Training organization	5.45	4.51 ± 0.51	0.12
A3‐B5‐C14 Satisfaction with Training Organization	5.45	4.54 ± 0.60	0.11
B6 Mastery of training content	18.68	4.64 ± 0.51	0.12
A3‐B6‐C15 Mastery of theoretical knowledge	6.05	4.68 ± 0.56	0.11
A3‐B6‐C16 Mastery of Operational Skills	5.24	4.71 ± 0.48	0.10
A3‐B6‐C17 Application of Comprehensive Skills	7.39	4.67 ± 0.53	0.11
B7 Radiation effects on tissues	26.99	4.60 ± 0.54	0.12
A3‐B7‐C18 humanistic concern	3.51	4.64 ± 0.58	0.13
A3‐B7‐C19 Behavioural Attitude Performance	1.77	4.80 ± 0.62	0.14
A3‐B7‐C20 Improvement of postcompetence	6.44	4.77 ± 0.61	0.12
A3‐B7‐C21 The influence of nursing quality	7.95	4.74 ± 0.64	0.15
A3‐B7‐C22 Organization benefit	3.38	4.39 ± 0.68	0.15
A3‐B7‐C23 Personal benefit	3.94	4.38 ± 0.64	0.14

Note

A first‐level indicators B secondary indicators C tri‐grade indicators.

### 3.2 Implementation results

According to different evaluation methods, each three‐level index was scored one by one. The score of each index was 100 points. The weighted cumulative comprehensive scoring method was used to multiply the average score of each index by the weight of the index to get the weighted score of the index (Table 5). All weighted scores were accumulated to get the total score of the index. The comprehensive score calculated in the study was 90.28.

**Table 5:** The Comprehensive score of the evaluation index system of pre‐job training effect for new nurses (score)

Tri‐grade index	Evaluation standard	Evaluation method	scores	Weight scores
A1‐B1‐C1 Policy and Planning	The hospital has a series of supporting policies, such as promotion, employment, tuition subsidies, implementation of the training plan for nurses and related documents for nurses' training.	Convening a Symposium on Relevant Managers、Access to relevant documents, etc.	100.00	2.11
A1‐B1‐C2 Teaching staff	Is the title, educational background, teaching age and professional level of the teaching staff reasonable?		100.00	3.62
A1‐B2‐C3 Teaching staff	Clear objectives: current nurses' knowledge of theoretical skills; training‐related attitudes	Questionnaire survey, examination system, on‐site examination, on‐site interview	100.00	3.94
A1‐B2‐C4 Training objectives	The degree of conformity between contents and training objectives; the systematic‐ness and continuity of curriculum contents; the content meets the needs of nurses and the depth and breadth of contents were reasonable.		92.00	4.57
A1‐B2‐C5 Training plan	It has pertinence and practicability; the training plan is focused and organized.		95.00	7.39
A1‐B2‐C6 Outline and textbooks	The outline is focused and organized; the textbooks reflect the latest achievements and progress.		90.00	2.03
A1‐B3‐C7 Training materials	Clinical Practice Diseases with Training Contents		100.00	4.20
A1‐B3‐C8 Training ground	All departments have training venues.		100.00	0.85
A1‐B3‐C9 Training Instruments and Equipment	Full preparation of training instruments and equipment; management mode and teaching atmosphere of training departments		100.00	0.50
A2‐B4‐C10 Course Hours Arrangement	Reasonable training schedule		90.00	3.51
A2‐B4‐C11Training contents	The degree of conformity between contents and training objectives; the systematicness and continuity of curriculum contents; the content meets the needs of nurses and the depth and breadth of contents were reasonable.		84.00	6.59
A2‐B4‐C12 Training teachers	Teaching Attitude; Professional Level; Teaching Skills and Teaching Methods; Teachers' Pre‐class Preparation		86.58	2.84
A2‐B4‐C13 Implementation of the plan	Implementation of the plan	Questionnaire investigation	80.19	2.93
A3‐B5‐C14 Satisfaction with Training Organization	Satisfaction with training organizations		86.95	4.74
A3‐B6‐C15 Mastery of theoretical knowledge	Theoretical knowledge	Examination system	88.49	5.35
A3‐B6‐C16 Mastery of Operational Skills	Operational skills	on‐site operation	93.41	4.90
A3‐B6‐C17 Application of Comprehensive Skills	Comprehensive skills	on‐site operation、Scenario simulation、Project research	75.57	5.58
A3‐B7‐C18 humanistic concern	Understand the feelings of patients and their families and be able to think transpositionally; respect patients, pay attention to protecting patients' privacy; pay attention to communication with critically ill and dying patients and their families.	360 degree evaluation、Questionnaire investigation、Key interview、guidance on‐the‐spot	85.81	3.01
A3‐B7‐C19 Behavioural Attitude Performance	Nursing Behavior Standard; Team Cooperation; Organizational Discipline; Professional Responsibility; Quality Nursing Service		80.19	1.42
A3‐B7‐C20 Improvement of postcompetence	Clinical Nursing Ability; Teaching Ability; Innovative Ability; Communication and Communication Ability; Emergency Response to Emergencies; Clinical Management Ability; Scientific Research Ability		82.86	5.34
A3‐B7‐C21 The influence of nursing quality	Incidence of adverse events; Ward safety management; Qualification rate of various quality indicators; Health Education		94.69	7.55
A3‐B7‐C22 Organization benefit	Nursing culture construction, patient complaint rate, patient satisfaction, nursing service quality, social and economic benefits and the incidence of nurse–patient disputes		100.00	3.38
A3‐B7‐C23 Personal benefit	Personal development (promotion of administrative position, teaching, becoming the backbone of clinical nursing), participation in discipline construction, achievements and job satisfaction		100.00	3.93
Comprehensive score				90.28

### 3.3 Application results

**Figure 3:**
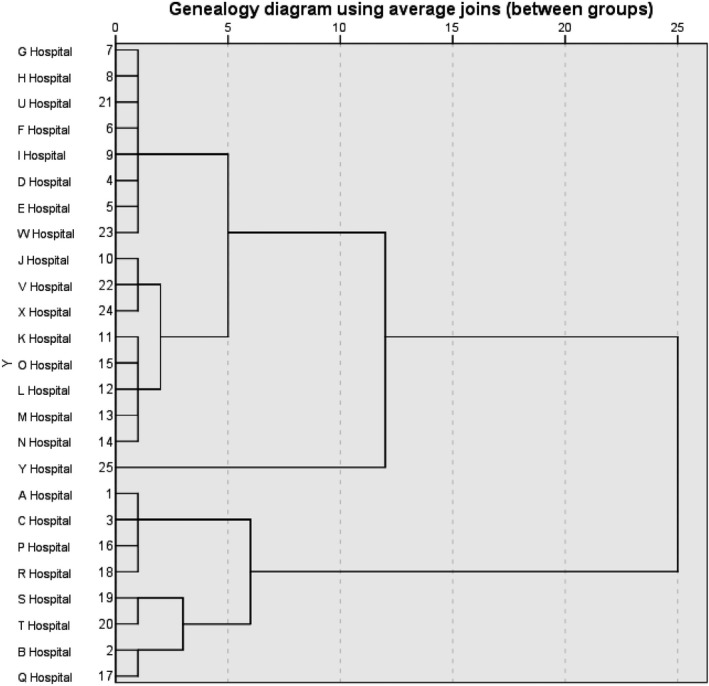
Tree Chart of Cluster Analysis for the Comprehensive Analysis of 25 Hospitals

Based on the results of on‐site evaluation, the overall situation of teaching and training in 25 hospitals is shown in Table 6. A cluster analysis of 25 hospitals showed that there were four levels of differences in teaching and training ability: the best, the better, the general and the worse. Hospitals A, B, C, P, Q, R, S and T were in one category, and the teaching and training abilities of the eight hospitals were the best; hospitals J, K, L, M, N, O, V and X were in one category; hospitals D, E, F, G, H, I, U and W were in one category; the teaching and training abilities of the eight hospitals were general, while hospital Y was in one category and the teaching and training abilities were relatively poor. The results of variance analysis showed that the distance between the four groups had significant statistical significance (*p < *.05). It showed that the evaluation index system of nursing teaching training based on the cluster analysis had a strong distinction and could evaluate the teaching training situation of the 25 hospitals better. The cluster analysis is shown in Figure 3.

**Table 6:** Empowerment Scores of Level I Index in 25 Hospital (Scores)

Name of hospital	Before training	Training process	After training	Total Scores
A hospital	26.21	15.87	47.19	88.28
B hospital	27.14	16.55	46.20	89.89
C hospital	26.49	14.29	45.19	87.97
D hospital	24.18	14.89	45.20	84.27
E hospital	25.19	14.10	44.70	83.99
F hospital	23.64	13.58	45.89	83.11
G hospital	22.16	14.06	46.69	82.91
H hospital	22.18	12.02	48.69	82.89
I hospital	21.49	12.54	48.19	82.22
J hospital	20.71	11.50	49.19	81.40
K hospital	19.18	10.98	49.69	79.85
L hospital	18.84	10.46	50.19	79.49
M hospital	18.50	9.94	50.69	79.13
N hospital	18.16	9.42	51.19	78.77
O hospital	19.87	10.25	49.56	79.68
P hospital	24.31	12.09	50.17	86.57
Q hospital	25.78	16.11	48.57	90.46
R hospital	19.99	17.29	49.72	87
S hospital	25.68	16.79	50.09	92.56
T hospital	28.01	17.49	47.36	92.86
U hospital	22.36	15.28	45.36	83
V hospital	21.79	14.58	44.89	81.26
W hospital	20.99	17.14	46.55	84.68
X hospital	19.85	16.55	44.31	80.71
Y hospital	17.94	14.98	43.12	76.04

## 4 DISCUSS

### 4.1 Reconstructing the nurse training evaluation model

Choosing an appropriate evaluation model as the theoretical framework can effectively guarantee the comprehensiveness and pertinence of the index setting. Suhayda et al. (2006) point out that the evaluation model is accountability‐oriented, emphasizing active evaluation and retrospective evaluation to guide the optimal design of training projects and ensure the quality of training evaluation. Classical evaluation models were used to guide the evaluation in different fields, such as continuing medical education (Moore et al., 2009), national administrative department (Frantz et al., 2015), bank (Matsumoto & Matthews, 2015), national defence and military industry (Torous et al., 2015), science and technology development (Bruce et al., 2015).

Based on classical models and a lot of theoretical analysis, foreign scholars have reconstructed their own models in various fields. For example, American Pross (2010) constructed a conceptual Pross evaluation model to promote excellence at the level of nursing education to systematically evaluate the educational benefits of complex nursing projects. Decker et al. (2011) reconstructed a physician training evaluation model, which was a conceptual model adapted from the Cocktail Model. The adjusted framework includes short‐term outcomes, such as changes in response to training and attitudes, knowledge or skills and long‐term outcomes, such as changes in physician‐related behaviour or patient outcomes in clinical practice. The study reconstructs a new evaluation model of nurse job training based on the comprehensive analysis of the Kirkpatrick model and CIPP model. The model covers multi‐level training evaluation strategies and generates abundant information. It could help researchers and managers conceptualize the potential training effect on nurses and select appropriate evaluation methods and tools in the specific organization or environment of training work.

### 4.2 Scientific‐ness and credibility of the evaluation index system

The effect of nurses' job training needs a scientific evaluation system to evaluate it comprehensively. This study combines the characteristics of Chinese nurse postmanagement, adhering to the evaluation concept of "starting from the end," from theoretical research to practical application and establishes an evaluation index system of nurse post‐training suitable for China's national conditions. The system adheres to the whole process evaluation path from pre‐training, to the training process, to post‐training. It covers the training needs investigation before training, the formulation of the training plan, whether the professional quality of teachers is in place in the training process, as well as the immediate indicators such as theory and operation skills after training, whether the short‐term indicators such as the improvement of comprehensive nursing quality bring social and economic benefits to hospitals or not. It is helpful to realize the long‐term indicators such as personal career development. It reflects the effect of nurses' job training from many angles, such as whether the level of clinical specialist skills is improved, whether the quality of comprehensive nursing is improved, whether the professional development and working ability of nurses were improved and whether the quality of the nursing service brings about the social and economic benefits of hospitals, etc. The evaluation index system is scientific.

The study strictly abides by the principle of the Delphi method application from the selection of experts, the formulation and distribution of questionnaires and the treatment of results, to ensure the reliability of the conclusions. Delphi consulting experts in the study were 26 experts from 11 tertiary hospitals and 6 experts from nursing colleges of 6 universities in China. They all have rich knowledge of medical education and medical management theory, rich working experience and certain academic authority in relevant fields. They have laid a good foundation for the reliability of index system construction. In the study, the positive coefficients of three rounds of experts were 100%, 100.0% and 96.88%, respectively. Most experts put forward amendments, reflecting the great attention and support given by experts to the study. The results showed that the expert authority coefficient was 0.82 and the higher the coefficient, the more authoritative and representative the expert inquiry results. The coordination coefficients were 0.889, 0.847 and 0.869, respectively, which showed that experts agree with each other, the trend was stable and the index weight structure had high reliability.

### 4.3 A weight analysis of the evaluation index

The results of the study showed that the weights of post‐training indicators were the highest (A3:52.98) and the weights of pre‐training indicators (A1:28.35) and training process indicators (A2:18.67) were the same, indicating that post‐training evaluation was the key part of training evaluation. Post‐training evaluation was to evaluate the final effect of training. It aimed to make managers know the advantages and disadvantages of training projects, understand the degree of achieving the expected training objectives and provide a basis for the formulation and implementation of training plans and projects. The second‐level index "radiation effect on the organization" (B8:27.86) has the largest weight among the same level index and the third‐level index "nursing quality to" (C27:9.44) has the largest weight. Nursing quality was one of the important symbols for measuring the quality of hospital service and the core and key of nursing management. Scientific quality evaluation was not only conducive to safeguarding the interests of patients, but also to the formulation of nursing quality improvement goals and programmes.

Under the secondary index of "to on the organization," the weight of nursing quality and postcompetence improvement was equal, 7.95 and 6.44, respectively, while the weight of behaviour and attitude performance was lower at 1.77. The reason for this may be that some consultants believe that behavioural attitudes were over‐expressed and that training is only part of it. Under the secondary index of "training and teaching," the weight of training content was the largest at 7.84. It indicated that the training content should be strengthened in the training settings and the teaching staff of nurses' post‐training should be standardized to provide efficient training support.

In the evaluation of nurses' training effectiveness, we need to take full account of the economic benefits, time and manpower benefits. Not all training needs to target all dimensions. We need to select appropriate evaluation indicators according to training content, training scale and training objectives. At the same time, different types of training will have different weight settings in different dimensions.

At present, the major hospitals spend more energy and financial resources on the evaluation indicators with lower relative weight and pay less attention to the results of clinical transformation of training such as organizational benefit. Future research should pay more attention to organizational effectiveness. The determination of weights is conducive to a better definition of "training effectiveness." Whether training is effective is no longer defined solely by the satisfaction of training organizations and the immediate grasp of training content and more attention should be paid to the effect of training migration; to a certain extent, the pre‐training and training process should be evaluated, but at the same time, when judging whether training is effective, the evaluation weights should be allocated reasonably so as to evaluate the results. The scientific nature lays the foundation.

### 4.4 The practicability of the evaluation index system

The pre‐training needs analysis of nurses is the first step in the whole process of training activities. It is the basis for formulating training plans, designing training programmes, implementing training activities and evaluating training effects. It is very important for nursing education and training. The training needs of ZK hospital were mainly based on the subjective judgement of the hospital management, focusing on the hospital needs and work needs, seldom considering the personal needs of employees, depriving employees of their autonomy in choosing training, thus reducing the enthusiasm of employees to participate in training. In the study, a pre‐job training needs questionnaire for new nurses was set up to determine the training needs of new nurses and establish training objectives, so as to promote long‐term planning and development of nurses, which is conducive to the overall human resources reserve and promotion of hospitals.

The index system constructed in the study regards pre‐training evaluation as the primary index and highlights its importance. It will guide medical institutions to attach importance to pre‐training evaluation, control the behaviour of evaluation objects and guide them to approach training objectives. During the training process, that is, three months after the training of new nurses, the feedback questionnaire of pre‐job training of new nurses and the evaluation form of clinical nursing teaching training plan were applied to timely understand the training feedback and correct and control the problematic items. Nemec (2018) calls the evaluation of training process as process control. It pays attention to whether the overall training process is standardized. It evaluates the training process from three main aspects: curriculum content, teachers and training‐related support. Different cycles were repeated to ensure the improvement of training effectiveness.

The results of Table 4 showed that the three‐level indicators "training teachers," "training content" and "plan implementation" score are lower when the system is used to evaluate the training process. The reason is that most of the nurses training teachers were backbone nurses in hospitals and few of them employ teachers from outside schools or from outside hospitals, which inevitably affects the teaching attitudes, teaching methods and skills, teachers' pre‐class preparation and the implementation of training plans of the training teachers. Therefore, the problem of nursing training teachers is a key issue related to the effectiveness of clinical nurses training and it is also an urgent problem to be solved at present. Zhou et al. (2016) point out that before each class, teachers must determine the teaching objectives, adjust the curriculum system timely and improve the implementation methods and evaluation strategies of nursing teaching. Hospitals should focus on "setting up platforms, gathering talents, stressing the introduction and training, creating mechanisms, increasing vitality and being the leader" and make every effort to promote the construction of a nursing talent team, so as to build a compound nursing team with both virtue and talent and excellent specialty. After transforming the index system into a questionnaire or a checklist, the study quantifies each three‐level index, together with a rigorous evaluation method, which can objectively and accurately reflect the problems in the training and provides a quantitative standard for the evaluation of the training quality of nurses. For the management department to use the evaluation system regularly, it can obtain a large amount of information about the training situation from multiple perspectives, grasp the situation dynamically and in a timely manner, clarify or adjust the training focus and input and provide a scientific basis for decision‐making. In applying the index system, we can select different medical institutions or clinical departments and carry out assessment, award evaluation and merit evaluation based on the evaluation results, which was conducive to improving the effectiveness of hospital training resources allocation.

### 4.5 Information‐based nursing training evaluation and the monitoring system

With the rapid development of global information technology, the development and application of hospital information systems have become one of the key aspects of current hospital development. Based on the practice of advanced hospitals at home and abroad and supported by hospital information systems, the author's hospital constructed and designed a nursing management information platform suitable for our hospital and applied it in clinical practice. The checklists and questionnaires designed in this study are embedded into NIS platform. All the evaluation processes are carried out via the information system. From the planning allocation, checking input, quality issues summary, quality indicators summary, quality indicators trend analysis, problem Plato analysis and related data derivation, the evaluation and analysis report of the effectiveness of nurses' training is automatically formed. The nursing information platform can realize an automatic statistical analysis of results, automatically generate index rates, histogram, polygraph, etc. At the same time, it can achieve year‐to‐year and ring‐to‐ring ratio. Nurses could grasp important data trends in a short time period and avoid the to of fragmented data and short‐term effect deviation.

The hospital introduced Jingyi Nursing Education Management Platform "512 Examination Education Network," which is based on cloud computing technology. It is a comprehensive teaching management platform for nursing department, head nurses and nurses. It can be used provided internet access is available. The 512 Examination Education Network covers comprehensive teaching management functions: teaching plan management, online training management, online examination management, training booking management, clinical operation assessment management, questionnaire survey management, statistical analysis management, skill file management and intern comprehensive management. It mainly solved two core problems in teaching management: 1. At present, the training time of nurses in China accounts for about 2% of the working time every year and the cost of manpower used in teaching was about 1 million yuan every year. Through the implementation of information systems, 50% of the manpower cost could be saved. 2. Current training methods take up a lot of time for nurses to work and take care of their families. Through information systems, nurses can choose training time independently and return the time to patients and family members to improve their job satisfaction. Relying on the information management platform, we established a modern and networked information management system to realize the “informationization” of clinical nursing teaching, which provided an objective, real‐time and dynamic data basis for the teaching evaluation of hospitals and promoted the teaching management of hospitals into a scientific and modern track.

## 5 LIMITATIONS

1. This study adopts convenient sampling rather than random sampling method and the surveys were mostly concentrated in East China, Central China and North China. The hospitals surveyed were all from tertiary and first‐class general hospitals. The training situation of hospitals at different levels in different regions of the country has not been comprehensively analysed and the evaluation results may be biased. The next research direction is to extend the evaluation index system of nurse post‐training and the information system of education quality management to the whole country and to implement unified evaluation criteria, methods, clinical application and supervision measures, so as to make horizontal and vertical comparisons of the effect of nursing training among different hospitals, departments and themselves and to provide scientific and standardized management of nursing training. Provide strong support.
2. Due to time and energy constraints, the content of formulating evaluation criteria and rules of the index system is not detailed enough. Some indicators may have some deviations when using qualitative evaluation methods. The evaluation criteria and rules should be further improved to make the evaluation tools more reliable.
3. Nurse training evaluation was single, ignoring the diversity of evaluation subjects. Most hospitals only evaluate the whole evaluation process by training managers and nursing officers, ignoring the value of self‐evaluation and other people's evaluation. Obviously, it will affect the objectivity and impartiality of evaluation. Therefore, it is suggested to form a multivariate evaluation team composed of training organization managers, training teachers, trained nurses, head nurses, colleagues (nurses and doctors) and patients, which can make a more comprehensive, scientific and objective evaluation of teaching activities from various perspectives.
4. The long‐term evaluation indicators set up in the study have lags and limitations; future research should further improve the long‐term evaluation indicators, retrospect the application of weak sub‐indicators and pay attention to the long‐term tracking of the application of training results; monitoring and evaluation of training activities are not achieved overnight and should be continuously monitored and periodically analysed to quantitatively reflect the effectiveness of the improvement of nursing training quality. The limitation of this study is that the index system is only validated in the clinical practice of our hospital. Future research should select several hospitals to apply the system to prove its feasibility and scientific‐ness.

### Understanding the lagging effect of the training effect in an all‐round way

Not all training projects can achieve immediate results, which can only be reflected after a certain amount of practice, which requires training to adhere to the principle of long‐term sustainability. Research such as that conducted by Eid and Quinn (2017) reveals three factors influencing the transfer of training: trainee characteristics, training courses and working environment. Relevant trainees' characteristics include attitudes to change, motivation, psychological processing ability, interpersonal skills and personality characteristics: curiosity, humility, seriousness, flexibility, intelligence and enthusiasm. Training projects, team learning and lectures were identified as relevant aspects of training courses. Work culture, work relationships and resources were all sub‐themes of the work environment category. In a systematic review, Beidas and Kendall (2010) suggested that the effectiveness of physician training was influenced by the quality of training, therapist variables, customer variables and organizational background. Decker et al. (2011) pointed out in a systematic review of the literature that no study assessed whether physicians' attitudes towards training affected patient outcomes. Although some researchers have only assessed attitudes at one point in time, we recommend assessing attitudes towards interventions or evidence‐based practices before, after and after training, as attitudes may change over time. Based on the system, it is recommended to continue to assess attitudes towards evidence‐based practices or specific interventions and to use measures with established reliability and preliminary validity data when feasible.

There were many factors hindering the transfer of training: nurses' behavioural awareness, the changing environment of training effect, whether the organizational cultural atmosphere is suitable for the application of training content, the change of thinking, the trainees' personal ability, the time of digestion and consolidation of trainees, the shortage of funds and the importance attached by hospital management. The lag of economic benefits makes management pay little attention to staff training. Departments should establish a mechanism for the transformation of training results to enable the trainees to transform what they have learned, thereby continuously improving their work efficiency. At the same time, in the definition of training effectiveness, we should not only expect positive results, but also offer critical evaluation results, which have a practical guiding role in optimizing teachers and curriculums and adjusting curriculum ratios. It is suggested that nursing managers should pay attention to the "huge fault" from learning level to behaviour result level and construct the driving force of training content transformation by means of monitoring, strengthening and rewarding, so as to ensure the effective transformation of the training content.

## 6 CONCLUSION

The evaluation index system of nurses' post‐training constructed in the study is scientific, practical and operable. It is of great significance for nursing managers to understand the effects of training and to improve training quality. It also provides a reference for training supervision of relevant health administrative departments. The index system is still in the initial stage of construction. Although its universality has been studied in some third‐class and first‐class general hospitals and has achieved remarkable results, it still needs to further expand the empirical research area.

## CONFLICT OF INTEREST

The authors declared no potential conflict of interest with respect to the research, authorship and/or publication of this article.

## AUTHORS’ CONTRIBUTIONS

The first author and the corresponding author made substantive intellectual contributions to the article. Linlin JIAO and Yuanda SUI conceptualized and designed the research and writing the paper. Guihua YANG collected and interpreted the data. Pulin WANG and Qiaofeng LI analysed the data. Junhong CHEN, Lili LIU and Chunling YANG interpreted the data and discussed core ideas. All authors had full access to the data and take responsibility for the integrity of the data and the accuracy of the data analyses.

## Data Availability

Data are available on reasonable request from the authors. Full data set available from the corresponding author at suiyuanda@126.com.
